# Impact of Genotype on EPA and DHA Status and Responsiveness to Increased Intakes

**DOI:** 10.3390/nu8030123

**Published:** 2016-03-02

**Authors:** Anne Marie Minihane

**Affiliations:** Department of Nutrition and Preventive Medicine, Norwich Medical School, BCRE, University of East Anglia (UEA), James Watson Road, Norwich NR4 7UQ, UK; a.minihane@uea.ac.uk; Tel.: +44-1603-592-389

**Keywords:** eicosapentaenoic acid, EPA, docosahexaenoic acid, DHA, long chain *n*-3 PUFA, genotype, *APOE*, *FADS*

## Abstract

At a population level, cardioprotective and cognitive actions of the fish oil (FO) derived long-chain *n*-3 polyunsaturated fatty acids (LC *n*-3 PUFAs) eicosapentaenoic acid (EPA) and docosahexaenoic acid (DHA) have been extensively demonstrated. In addition to dietary intake, which is limited for many individuals, EPA and DHA status is dependent on the efficiency of their biosynthesis from α-linolenic acid. Gender and common gene variants have been identified as influencing the rate-limiting desaturase and elongase enzymes. Response to a particular intake or status is also highly heterogeneous and likely influenced by genetic variants which impact on EPA and DHA metabolism and tissue partitioning, transcription factor activity, or physiological end-point regulation. Here, available literature relating genotype to tissue LC *n*-3 PUFA status and response to FO intervention is considered. It is concluded that the available evidence is relatively limited, with much of the variability unexplained, though *APOE* and *FADS* genotypes are emerging as being important. Although genotype × LC *n*-3 PUFA interactions have been described for a number of phenotypes, few have been confirmed in independent studies. A more comprehensive understanding of the genetic, physiological and behavioural modulators of EPA and DHA status and response to intervention is needed to allow refinement of current dietary LC *n*-3 PUFA recommendations and stratification of advice to “vulnerable” and responsive subgroups.

## 1. Introduction

Although randomised controlled trials (RCT) are inconsistent [1,2,3,4], there is a large body of cell, animal and human prospective cohort data demonstrating the cardiovascular and cognitive benefits of increased fish consumption and eicosapentaenoic acid (EPA) and docosahexaenoic acid (DHA) intake and tissue status, with underlying physiological and molecular mechanisms identified [5,6,7,8,9]. Such evidence has translated into typical national and international recommended intakes of >500 mg of EPA + DHA per day in the general population to improve cardiovascular health, >1 g EPA + DHA per day for the secondary prevention of CVD, with >200 mg DHA per day recommended in pregnancy [10,11,12]. Despite the provision of such generic recommended intakes there is a wide recognition that intake-independent EPA and DHA status and response to increased EPA and DHA intakes is highly variable, with the aetiology of this heterogeneity poorly understood.

**Figure 1:**
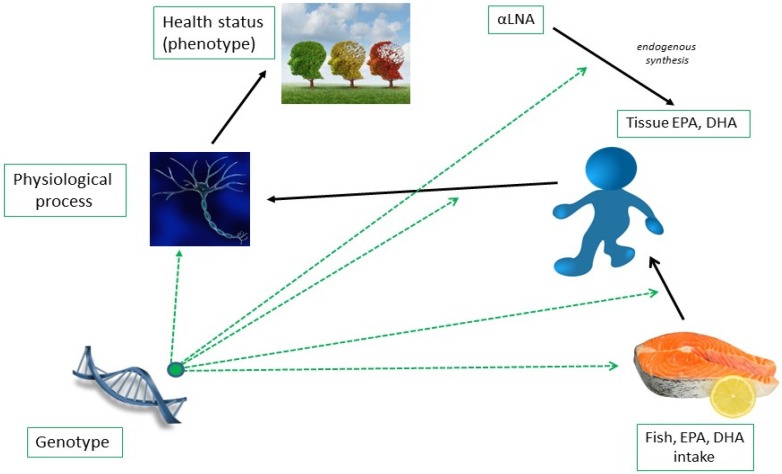
Overview of the potential of genotype to influence EPA and DHA status and responsiveness.

Unlike typical nutrients, which cannot be synthesised *in vivo*, EPA and DHA can to some extent be synthesised from the precursor plant derived shorter chain *n*-3 fatty acids, α-linolenic acid (αLNA) [13,14], with gender [15] and variants [16] in the rate limiting enzymes of the biosynthetic pathway emerging as important determinants of the biosynthetic efficiency (Figure 1). Genotype is also known to be important in taste and sensory perception and therefore food preference and intake [17,18]. In many populations oily fish is poorly tolerated relative to other foods, and regularly consumed by only a minority of the population [19]. Although completely unknown it is likely that genotype is an important modulator of oily fish taste sensitivity and consumption and therefore EPA and DHA intake. Once consumed the absorption of EPA and DHA, their subsequent tissue and cellular partitioning, and their oxidation or metabolism into lipid derived bioactivities, is variable and likely genotype dependent. Finally the impact of a particular tissue/cell EPA and DHA (or their metabolite) status on cell signalling, physiological processes and ultimately health biomarkers or clinical end-points will also be modulated be numerous variants in genes encoding, fatty acid responsive transcription factors and other cell signalling molecules and their physiological targets.

There are numerous single reports in the literature of the impact of individual gene variants on LC *n*-3 PUFA responsiveness. Rather than attempt to be exhaustive and report on all of these findings, the majority of which require confirmation in independent studies, the review will largely focus on a select number of genes and genotypes which have been relatively consistently shown to regulate EPA and DHA status or responsiveness. Such genotypes may in the future be useful in the targeting of specific EPA and DHA recommendations towards individuals likely to be deficient and responsive.

## 2. Genetic Determinants of EPA and DHA Biosynthesis and Status

Familial aggregation analysis indicates that 40%–70% of (red blood cell (RBC)) fatty acid status is heritable [20]. In the Framingham Heart Study, 73% of the variability in the RBC omega-3 index (EPA + DHA as a % total of total fatty acids (FA)) was explained by participant characteristics added to the regression model, which included heritability (24%), EPA + DHA intake (25%), and fish oil supplementation (15%) [21].

**Figure 2:**
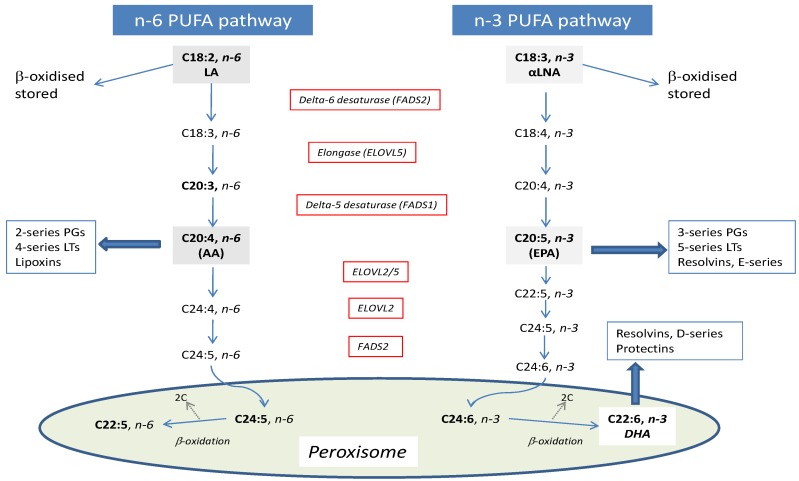
Long chain polyunsaturated fatty acid biosynthetic pathway.

The endogenous synthesis of the LC PUFA, arachidonic acid (AA), and EPA/DHA occurs mainly in the liver in humans, via a common series of desaturation and elongation reactions (Figure 2), with delta-5 desaturase (D5DS) and delta-6 desaturase (D6DS) encoded by *FADS1* and *FADS2* genes representing major regulatory steps. This pathway is the main source of tissue EPA and DHA in those who consume little or no seafood or fish oil supplements. The efficiency of the pathway is inherently low in humans, with an estimated conversion of αLNA to EPA of 0.2%–6% and <0.1% for DHA [13], and therefore any changes in bioconversion efficiency have potentially large impacts on LC PUFA status.

The *FADSs* genes located as a head-to-head cluster on chromosome 11 (11q12.2-q13.1) [22] are highly polymorphic with 4391 variants, predominately single nucleotide polymorphisms (SNP), described in the *National Center for Biotechnology Information* dbSNP database [23], 217 of which are missense resulting in amino acid changes in the D5DS and D6DS proteins. In 2006, 18 SNPs in the gene cluster were genotyped in 727 adults in the *German Centre of the European Community Respiratory Health Survey* [24]. All haplotypes (grouping of variants) which included the minor alleles were associated with increases in αLNA and linoleic acid (LA) and decreases in γ-linolenic acid, AA, EPA and *n*-3 docosapentaenoic acid (DPA), with no significant impact on DHA or *n*-6 DPA evident. A 5-locus haplotype explained 27.7%, 5.2% and 1.4% of the variability in AA, EPA and DHA levels respectively. Interestingly this haplotype was associated with a greater than 50% lower incidence of the chronic inflammatory conditions atopic eczema and allergic rhinitis, which may be due to the lower availability of AA for cyclooxygenation to the strong pro-inflammatory 2-series prostaglandins and 4-series leukotrienes. Over the last decade, and taking a similar candidate gene approach, these initial observations of the association between *FADS1-FADS2* SNPs and haplotypes and D5DS and D6DS activities, plasma, tissue and breast milk fatty acid composition and the incidence of diseases with chronic inflammatory components have been confirmed in subsequent studies [25,26,27,28,29,30,31,32]. In the *Verona Heart Study*, a strong association with coronary artery disease was evident, with an incidence of 84% *versus* 66% in individuals with 6–7 *versus* 2–3 risk alleles [27].

DHA status during pregnancy influences infant growth and development, with breast feeding generally recommended till at least 6 month post-partum. Xie *et al.*, demonstrated lower breast milk ARA, EPA, *n*-3 DPA and DHA in individuals homozygous for *FADS1-FADS2* minor alleles [30]. In Danish infants the impact of breast feeding, fish intake and *FADS* genotype on RBC DHA status at 9 m and 3 years of age was assessed [33]. Collectively these variables explained 25% of the variation in status at 9 m (mean DHA of 6.6% of total FA%). Homozygous carriers of the minor allele of rs1535 had a DHA increase of 1.8 FA% whereas minor allele carriers of rs174448 and rs174575 had a decrease of 1.1 and 2.0 FA%, relative to the wild-type genotype. Interestingly further analysis indicated that about a 50 g fish intake would be needed to mitigate the impact of having only two DHA “raising” allele relative to five, highlighting the importance of *FADS* genotype on infant DHA status against a background of limited intake. In the *Koala Birth Cohort* the observation of an association between low maternal DHA intake with a reduced birth weight only in *FADS* minor allele carriers [34], again reinforces the importance of DHA intake in maternal-infant nutrition against a *FADS* genotype background associated with reduced endogenous synthesis.

Along with candidate gene approaches, untargeted unbiased genome wide association study (GWAS) has approaches have also identified the *FADS1-3* and also *elongase* (*ELOVL)* genes, as being associated with LC PUFA status [35,36,37,38]. In five population-based cohorts comprising approximately 900 individual, and consistent with the initial observation of Schaeffer *et al.*, published in 2006 [24], variant alleles of *FADS1* and *FADS2* were associated with higher levels of αLNA and lower levels of EPA and DPA, with variant alleles of *ELOVL2* associated with higher EPA and DPA and lower DHA, suggesting a decreased elongation of DPA to DHA [35]. *ELOVL2* encodes elongase 2 which is critical in the elongation of DPA to DHA [39] (Figure 2) The associations were independent of fatty fish intake, with an absence of interaction consistent with the *Koala Birth Cohort* who observed similar slopes of plasma EPA and DHA in those with 0, 1 or 2 minor *FADS1-FADS2* alleles [40].

GWAS have highlighted the physiological significance of variation in the *FADS* locus, with associations with plasma total cholesterol (TC), LDL-cholesterol (LDL-C), triglycerides (TG) and PUFA composition reported [36,41,42]. In a recent GWAS analysis to investigate genetic signatures of diet and climate adaptation in Greenland Inuits, who have a high LC *n*-3 PUFA intake, *FADS* was the strongest locus associated with height, weight, growth hormone regulation and membrane fatty acid composition [43].

In addition to observational analysis, the impact of *FADS* variants on response to EPA and DHA supplementation has been examined. In the *MARINA* RCT, the *FADS* rs174537 genotype interacted with treatment to determine D5DS activity; however no genotype × treatment interaction was evident for RBC EPA% and DHA%, which the authors suggested may be due to insufficient power [44]. In the same RCT *ELOVL2* gene SNPs did emerge as modulators of the TG response. After the 1.8 g/day dose, minor allele carriers had approximately 30% higher proportions of EPA and 9% higher DHA than non-carriers [45].

Although *FADS* and *elongase* variants have emerged as strong determinants of LC *n*-3 PUFA and some information is available as to factors which may modulate genotype-fatty acid status [46] granularity is still lacking regarding the relative effect size in various populations and the likely influences of factors such as ethnicity and habitual intake on the penetrance of genotype. Furthermore in the studies reported thus far associations between a large number of individual SNPs in *FADS* and *elongases* genes and fatty acid status and “health” outcomes have been observed many of which exist in a highly preserved linkage disequilibrium (LD) block and therefore co-inherited. The question remains as to which are the actual functional SNPs and what is the molecular aetiology of the effect of the variant on EPA and DHA status. In a recent seminal paper, Wang and co-workers conducted an analysis of the association between six *FADS* SNPs and the lipidomic profile, *FADS1-3* gene expression and protein levels in 154 human liver samples All six allele were associated with *FADS1* but not *FADS2* and 3 gene expression and also FADS1 protein levels, indicating *FADS 1* is the causal gene [38]. Furthermore they identified that among 42 highly linked SNPs, 29 were in the transcription factor (TF) binding sites of the locus. Although it is unclear exactly which SNP(s) is causal for the altered FADS1 gene function, and the exact nature of how the SNP influences TF interaction with *FADS1*, such mechanistic insights add considerable credibility to the observed association between *FADS* and EPA and DHA. Further such work will lead to the identification of the most significant variant(s) which could be used to, identify individuals at risk of compromised EPA and DHA status, and target recommendations for additional intakes.

## 3. Impact of *APOE* Genotype on EPA and DHA Status and the Response to Fish Oil Intervention

Apolipoprotein E, first described as a component of circulating lipoproteins and a modulator of their metabolism [47,48], has subsequently been identified as the main lipid transporter in the central nervous system (CNS). Two missense SNPs in the *APOE* gene on chromosome 19, result in three apoE protein isoforms, namely apoE2, apoE3 and apoE4 which are distinguished by cysteine to arginine substitutions at positions 112 and 158 in the protein: apo2 contains cysteine at both positions, apoE3 contains cysteine at 112 and arginine at 158, with apoE4 containing arginine at both sites [47,49]. Although not in the receptor or lipid binding regions, the amino changes influence salt bridge formation between the *N*- and *C*-terminals domains of the protein which have profound impacts on receptor biding activities, lipoprotein preference and apoE stability and ultimately tissue protein concentrations [49]. *APOE4* carriers have been inconsistently shown to be at higher risk of cardiovascular diseases [50,51], with a variable penetrance attributed to modifiers such as, saturated fat [52] and cholesterol [53] intakes, and smoking status [54]. *APOE* genotype has emerged as the strongest identified common genetic predictor of longevity [55,56]. In the Genetics of Healthy Ageing Study, the prevalence of the *APOE4* allele was 6.8% in nonagenarians (90–99 years old), compared to 12.7% in matched control (55–75 years old), with *APOE4* carriers having a 50% lower chance (odds ratio (OR) = 0.48, 95% CI, 0.42–0.55) of reaching age 90 years compared to non-*APOE4* carriers [55]. This reduced longevity reflects the effect of genotype on risk of age-related cognitive decline and Alzheimer’s disease (AD), with *APOE3/E4* (20% Caucasians) and *APOE4/E4* (1%–2% Caucasians) individuals at approximately 4- and 15-fold increased risk of AD with a 10–20 years earlier age of onset [57].

Numerous potential mechanisms have been proposed to explain this association with cardiovascular and cognitive health, including an impact of *APOE* genotype on LC *n*-3 PUFA status and response of risk biomarkers to LC *n*-3 PUFA intakes. Brain tissue is highly enriched in DHA, indicating its essentiality to neuronal function. Although not investigated prospectively or as a primary study aim, a limited number of human studies have retrospectively reported that the cognitive benefits associated with DHA/fish intake were absent or lower in *APOE4* carriers [58,59,60]. For example in the *Cardiovascular Health Cognition Study*, Huang and co-workers reported that in the cohort as a whole consumption of oily fish more than twice per week was associated with a reduction in risk of AD by 41%, but stratification by *APOE* showed this effect to be selective to those without the *APOE4* allele [55]. Supplementation with DHA for 18 m did not slow the rate of cognitive decline in patients with mild to moderate Alzheimer disease [59]. Retrospective subgroup analysis indicated some cognitive benefits in non-*E4* carriers consistent with the epidemiological data. Variability in LC *n*-3 PUFA metabolism according to *APOE* genotype is likely to partly explain the differential cognitive response to increased DHA intake and status. In the *Three-City Cohort* of older adults, plasma EPA and DHA proportions did not differ according to *APOE* genotype but the association between fish consumption and plasma DHA was weaker in *APOE4* carriers. This is consistent with the *SATGENE* intervention, in which participants were prospectively recruited by *APOE* genotype. Following supplementation with DHA (3.5 g per day) for 8 weeks, a 21% lower plasma phospholipid DHA enrichment was observed in overweight *APOE3/E4* relative to *APOE3/E3* individuals [61]. *APOE4* carriers have lower plasma concentrations of apoE, which is in part attributed to lower hepatic apoE recycling, and apoE4 is preferentially associated with VLDL rather than HDL, with the opposite true for apoE3 [62]. Hence, although the aetiology of differential cognitive and plasma DHA responses to changes in DHA intake is currently poorly understood these *APOE* mediated differences in overall protein concentrations and lipoprotein partitioning together with a higher β-oxidation of DHA and lower brain uptake of a (14C)-DHA uptake associated with the *APOE4* allele [63,64], are likely to be involved.

Brain DHA is sourced from the systemic circulation with transport across the BBB involving a number of traditional members of the LDL-receptor family which use apoE as a ligand [65], along with the recently identified Mfsd2a [66]. The impact of *APOE* genotype on the expression and function of these transporters is currently unknown.

Although not fully consistent *APOE* genotype has also been shown to influence the plasma lipid response to EPA and DHA intervention, with indications of greater responsiveness in *APOE4* carriers, which may in part reflect the above described impact of genotype on fatty acid partitioning or the higher baseline LDL-C and TG evident in *APOE4* individuals [67,68,69,70,71,72]. In the *SATGENE* intervention a genotype × diet interaction was evident for plasma TG, with 17% and 30% decreases in *APOE3/E3* and *APOE3/E4* individuals after the high fat-high saturated fat-DHA relative to the low-fat diet [67]. A greater LC *n*-3 PUFA induced increase in adipose tissue lipoprotein lipase expression may in part explain the greater TG lowering in *APOE4* carriers [73], with endothelial associated LPL being the main enzyme responsible for the hydrolysis of circulating TG-rich lipoproteins. There is some earlier evidence of a borderline significant LDL-cholesterol raising effect of DHA in *APOE4* carriers in those with modest hypertrigylceridaemia [71] which was not evident in later studies in normolipdaemic individuals [67] or using more moderate intervention doses [68]. In a cross-sectional analysis in 137,000 individuals Harris *et al.*, observed no association between RBC omega-3 index and plasma LDL-C concentrations [69].

## 4. Genetic Variability and the Triglyceride Response to EPA and DHA

Elevated fasting and postprandial TGs are highly clinically significant CVD risk factors, of ever increasing prevalence, due to their strong association with adiposity and a loss if insulin sensitivity [74]. Perhaps the best described effect of EPA and DHA supplementation is its hypotrigylceridaemic actions, with the *American Heart Association* recommending intakes of 2–4 g per day as a TG lowering strategy [12]. But the TG response to increased EPA and DHA intakes is highly variable. In the *FINGEN* trial, although an overall significant impact of intervention was observed, no TG lowering was evident in 118 out of 312 participants in response to the higher dose [68,75]. As yet the genetic basis for this variable TG response is poorly understood. In addition to *APOE* and *FADS* genotype described above effects of variants in a number of genes involved in fatty acid metabolism and in LC *n*-3 responsive transcription factors have been described [27,76,77,78,79,80], the majority of which have not yet been confirmed in independent studies. For example in the 208 adults in the *Quebec City* Cohort, who were supplemented with ~3 g EPA + DHA per day, SNPs in two lipogenic genes, namely *ATP citrate lyase* (*ACLY*) and *acetyl-CoA carboxylase* (*ACACA*) explained 8% of the TG response [76]. In the same cohort and using an untargeted GWAS approach, SNP frequencies were compared in responders and non-responders. Although no SNP were identified using the calculated threshold for statistical significance (*p* < 1.87 × 10^−8^), 13 variants emerged using a more lenient statistically suggestive *p* value (*p* < 1 × 10^−5^). A genetic risk score (GRS) constructed using these SNPs explained 22% of the variation in the TG response to supplementation, with this GRS explaining a much more modest proportion of variation in the TG response in the confirmatory *FINGEN* cohort [81].

## 5. Closing Remarks

Dietary recommendations typically suggest an intake of EPA plus DHA of at least 500 mg per day. It is likely that higher intakes are needed to meaningfully modify many of the responsive CVD risk factors, providing some justification for increasing the current recommended intakes. However EPA and DHA supply and sustainability is an issue, with current sources, almost exclusively derived from fish, providing only 40% of what is needed in order for individuals globally to consume 500 mg per day [82]. The heterogeneity in response and this issue of supply provides a rationale to stratify advice to responsive individuals. But current understanding of the determinants of response is incomplete, with only a proportion of the genetic contribution identified and fully substantiated, and the mechanistic basis of identified genotype × LC *n*-3 PUFA interactions poorly understood. Such information must be gained from adequately powered “fit-for-purpose” studies, avoiding under-powered investigations which may be associated with spurious conclusions. Research to date has largely employed a candidate gene type approaches, with a future wider use of untargeted approaches such as GWAS or sequencing, in combination with a sensitive capture of EPA and DHA intake or status, needed to identify novel genetic modulators of EPA and DHA responses. 

## 6. Conclusions

Common gene variants are likely to be an important determinant of EPA and DHA status and associated physiological impacts. In the future, and with a more robust knowledge base, it is hoped that genotype could contribute to the targeting of dietary advice with for example increased intakes recommended in pregnancy to those with a *FADS-elongase* genetic profile indicative of a compromised EPA and DHA endogenous biosynthesis, or to *APOE4* individuals who may be likely to particularly benefit from the cognitive or TG lowering benefits.
